# Evaluating the Efficacy of Topical Phenytoin in the Healing of Neuropathic Diabetic Foot Ulcers: A Comparative Study

**DOI:** 10.7759/cureus.63282

**Published:** 2024-06-27

**Authors:** Tabana C, Shaheen Banu A, Saravanakumar Ganesan

**Affiliations:** 1 General Surgery, Government Sivagangai Medical College & Hospital, Sivagangai, IND

**Keywords:** conventional dressings, mild infection, healing outcomes, neuropathic diabetic foot ulcers, topical phenytoin

## Abstract

Objective: This study aims to observe the outcomes of topical phenytoin treatment in healing neuropathic diabetic foot ulcers with mild infection and compare these outcomes with those obtained from conventional dressing methods.

Methods: A retrospective observational study was conducted by reviewing the medical records of patients with neuropathic diabetic foot ulcers treated at a tertiary care center from 2015 to 2020. Two groups were identified: (1) patients treated with topical phenytoin (for ulcers measuring less than 5 cm, a dosage of 100 mg was used; for ulcers measuring between 5 cm and 9 cm, a dosage of 150 mg was used; for ulcers measuring between 10 cm and 15 cm, a dosage of 200 mg was used; and for ulcers measuring greater than 15 cm, a dosage of 300 mg was used. The tablets were crushed and dispersed before administration) and (2) those were treated with conventional dressings (the conventional method includes wound wash with 0.9 normal saline and betadine solution with application of sterile gauze dressing). Data on wound healing rate, time to achieve complete healing, and recurrence rates were collected.

Results: The study included 120 patients, with 60 receiving topical phenytoin and 60 receiving conventional dressings. Preliminary findings indicated that the topical phenytoin group experienced a 27 (45%) reduction in ulcer size by week four, compared to a 15 (25%) reduction in the conventional group. The median time to complete healing was significantly shorter in the phenytoin group (eight weeks) compared to the conventional dressing group (12 weeks; p < 0.05). Additionally, granulation tissue appeared earlier in the phenytoin group, with an average onset of 10 days, compared to 18 days in the conventional group (p < 0.01). The incidence of ulcer recurrence was lower in the phenytoin group (6, 10%) compared to the conventional group (18, 30%; p < 0.05).

Conclusions: Topical phenytoin demonstrated a promising enhancement in the healing of mildly infected neuropathic diabetic foot ulcers compared to conventional dressings. Further studies are recommended to substantiate these findings and explore the mechanisms underlying the observed benefits.

## Introduction

Diabetes mellitus (DM) stands as a predominant metabolic disorder of the 21st century, escalating rapidly due to factors such as obesity, sedentary lifestyles, and increasing urbanization. Among the myriad complications associated with diabetes, diabetic foot ulcers (DFUs) represent a critical concern, leading to severe morbidity and, in some cases, amputation [1]. The pathogenesis of DFUs involves a complex interplay of hyperglycemia, peripheral neuropathy, arterial insufficiency, and heightened susceptibility to infection, each contributing to the chronicity of these wounds [2].

Conventional management strategies for DFUs include meticulous wound care, infection control, and optimizing metabolic control. However, the delay in wound healing associated with these ulcers often results in significant economic and personal burdens, affecting patient quality of life and increasing healthcare costs. It is estimated that approximately 15% of patients with diabetes will experience a foot ulcer in their lifetime, with a significant number requiring subsequent amputation [2].

To improve wound healing in DFUs, alternative therapies have been investigated in light of these obstacles. In this regard, phenytoin, a widely prescribed anticonvulsant, has demonstrated potential. Phenytoin, which has been employed historically to treat a range of ulcer types, such as pressure ulcers and venous stasis ulcers, promotes wound healing by inhibiting collagenase activity, stimulating fibroblast proliferation, and promoting collagen deposition [3-6]. This ultimately facilitates the formation of granulations. Prior research has established that topical phenytoin is effective at promoting wound healing; therefore, it is plausible to consider it for comparative analyses with conventional treatments [1,7-9].

While there is a substantial body of research on the use of topical phenytoin for various types of ulcers, including DFUs, this study aims to provide additional evidence specifically focused on neuropathic DFUs with mild infections. Numerous studies have demonstrated the potential benefits of phenytoin in promoting wound healing and reducing infection. However, recent real-world studies seek to enhance the current knowledge base by evaluating the clinical outcomes associated with topical phenytoin compared to conventional dressing methods.

## Materials and methods

Study design and setting

Medical records from the Government Sivagangai Medical College & Hospital were analyzed for this retrospective observational study. The consultant of the unit is a podiatric surgeon; therefore, all cases of foot ulcers were admitted and treated in this specific unit of the surgery department. The objective of this study was to compare the efficacy of topical phenytoin application to traditional dressing methods when it comes to the treatment of moderate infection in neuropathic DFUs. The research obtained approval (5088/ME5/2022), and then cases were identified with grade 1b neuropathic DFUs, which are classified by the Texas wound classification system from the hospital records. The sample included patients treated between 2015 and 2020.

The sample size was calculated using the following formula: n=2×(Z_α/2_​+Z_β​_)^2^×(_p1_​×(1−_p1_​)+_p2_​×(1−_p2​_))​/(p1​−p2​)^2^, where Z_α/2​_ and 𝑍_𝛽_ correspond to the desired level of significance (α) and power (1 - β), respectively; p1​ and p2​ are the estimated proportions of the event in the two groups being compared.

From the records, 120 patients meeting these criteria were identified: 60 who received topical phenytoin and 60 who received conventional dressings. This retrospective identification ensured that a consistent cohort of patients was obtained, enabling a targeted comparison between the two treatment methods. For the topical phenytoin group, phenytoin was used in the form of tablets. They were crushed and dispersed directly over the ulcer, with the doses depending on the size of the ulcer. For ulcers measuring less than 5 cm, a dosage of 100 mg was used. For ulcers measuring between 5 cm and 9 cm, a dosage of 150 mg was used. For ulcers measuring between 10 cm and 15 cm, a dosage of 200 mg was used. For ulcers measuring greater than 15 cm, a dosage of 300 mg was used. Since most of the ulcers were of a smaller grade and size, the dressings were done once every three days. Phenytoin was applied every third day when the dressings were changed. The conventional dressings group involved standard wound care practices, including wound cleaning and debridement with 0.9% normal saline and betadine solution, moist wound dressings with sterile gauze, off-loading to reduce pressure on the ulcer, infection control with antibiotics if necessary, and patient education on proper foot care.

The patients ranged in age from 30 to 60 years. Patients who met the inclusion criteria had diabetes under control, as indicated by fasting and postprandial blood sugar levels both falling below 140 mg/dL and 200 mg/dL, respectively. To be enrolled in the study, the participants were obliged to provide informed consent. The patients who met the exclusion criteria included those who had uncontrolled diabetes or hypertension, ulcer grades lower than 1b, peripheral artery disease, coronary artery disease, osteomyelitis, chronic kidney disease, steroid therapy, a body mass index (BMI) greater than 30, or an abnormal ankle-brachial index. By employing this methodology, a consistent cohort of patients was obtained to conduct a targeted examination of the disparities between applied phenytoin and conventional dressing methods when it comes to managing moderate infections in DFUs.

Data collection

Demographic information (e.g., sex), ulcer characteristics (e.g., size, location), treatment particulars (e.g., dressing type, application frequency), and clinical outcomes (e.g., rate of ulcer healing, time to complete healing, recurrence) were acquired through an examination of patient records. Furthermore, data were collected regarding the administration of systemic antibiotics and any adverse effects observed throughout treatment. The extent of the ulcer upon day 1 and day 15 of treatment constituted the primary data points from which the reduction rate was computed. The application of phenytoin was simple, cost-effective, and additionally increased patient compliance, making it a preferred option for patients who consented.

Statistical analysis

The researchers conducted comparative analyses of the phenytoin and conventional dressing groups by utilizing the Student's t-test for continuous variables and the Chi-square test for categorical variables. A p-value of less than 0.05 was assumed to indicate statistical significance. Every analysis was performed utilizing IBM SPSS Statistics for Windows, Version 26 (Released 2019; IBM Corp., Armonk, New York).

## Results

A comprehensive analysis of the clinical attributes and demographic data of the two treatment groups is presented in Table 1.

**Table 1:** Clinical and demographic attributes of the phenytoin group of the study p-values < 0.05 are considered statistically significant SD: standard deviation; BMI: body mass index; NA: not applicable

Characteristics	Phenytoin Group (n=60)	Conventional Dressing Group (n=60)	p-value
Age (years, mean ± SD)	48.6 ± 5.2	49.2 ± 5.4	0.45
Gender (male/female)	30/30	29/31	0.88
Duration of diabetes (years, mean ± SD)	>5 years (avg)	>5 years (avg)	NA
BMI (mean ± SD)	27.5 ± 1.8	27.5 ± 1.7	0.99
Family history of diabetes n (%)	48 (80%)	50 (83%)	0.67

In contrast to the conventional dressing group, which had an average age of 49.2 years (±5.4), the phenytoin group expressed an average age of 48.6 years (±5.2). The gender distribution in the phenytoin group is balanced, with 30 males and 30 females, and the conventional dressing group has an identical gender distribution, with 29 males and 31 females. Patients with diabetes for a duration exceeding five years comprised the majority of both cohorts. The mean BMI for both cohorts remained constant at 27.5 (with a deviation of 1.8 in the phenytoin group and 1.7 in the conventional group), indicating that both groups were moderately overweight. A total of 48 (80%) patients in the phenytoin group and 50 (83%) in the conventional dressing group reported a family history of diabetes, indicating a substantial genetic predisposition within the study population. Table 2 delineates the effectiveness of the two healing approaches under review.

**Table 2:** Wound healing outcomes SD: standard deviation

Outcome	Phenytoin Group (n=60)	Conventional Dressing Group (n=60)	p-value
Initial ulcer size (cm², mean ± SD)	4.2 ± 1.0	4.1 ± 1.1	0.76
Reduction of ulcer size >75% n (%)	40 (66.67%)	16 (26.67%)	<0.01
Onset of granulation tissue (days, mean)	6	15	<0.01

Both groups started with nearly identical ulcer sizes; the average measurements were 4.2 cm² (with a standard deviation of ±1.0) for those treated with phenytoin, and marginally smaller at 4.1 cm² (with a standard deviation of ±1.1) for the group using conventional dressings. Remarkably, a significantly higher percentage of participants treated with phenytoin (40, 66.67%) achieved a reduction in ulcer size of over 75%, compared to only 16 (26.67%) in the group using conventional methods. In terms of granulation tissue, which signals critical progress in wound recovery, its appearance was accelerated in the phenytoin group, manifesting in just six days on average. This is considerably faster than the 15 days typically required in the conventional treatment group. The adverse events and corresponding outcomes of wound cultures are detailed in Table 3.

**Table 3:** Adverse events and wound culture outcomes p-values < 0.05 are considered statistically significant

Outcome	Phenytoin Group (n=60)	Conventional Dressing Group (n=60)	p-value
Mild itching n (%)	3 (5%)	1 (1.67%)	0.31
Initial culture positive n (%)	36 (60%)	28 (46.66%)	0.18
Culture positive on day 15 n (%)	14 (23.33%)	10 (16.66%)	0.29

Only three (5%) individuals in the phenytoin group and one (1.67%) in the conventional group reported experiencing mild irritation. The percentage of initial cultures that were positive was 36 (60%) in the phenytoin group, while it was 28 (46.66%) in the conventional dressing group.

**Figure 1:**
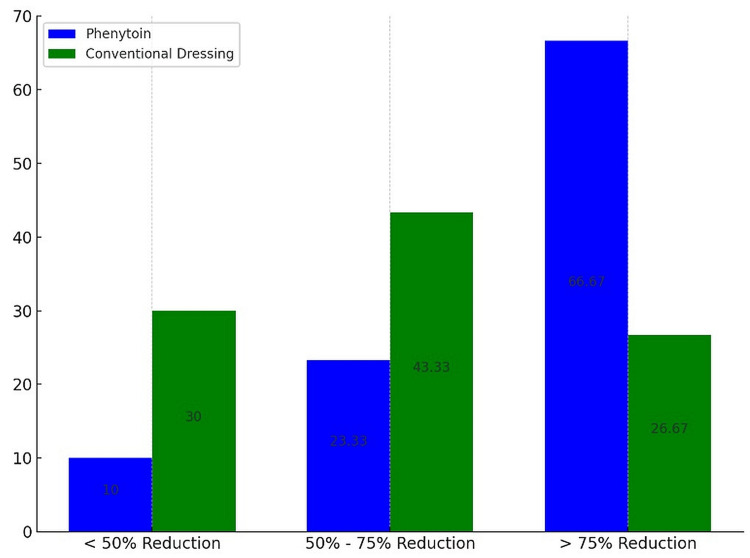
Comparative efficacy of ulcer size reduction: phenytoin vs. conventional dressing Phenytoin group (P): topical application of phenytoin; conventional dressing group (C): standard wound dressing methods Y axis: number of patients

**Figure 2:**
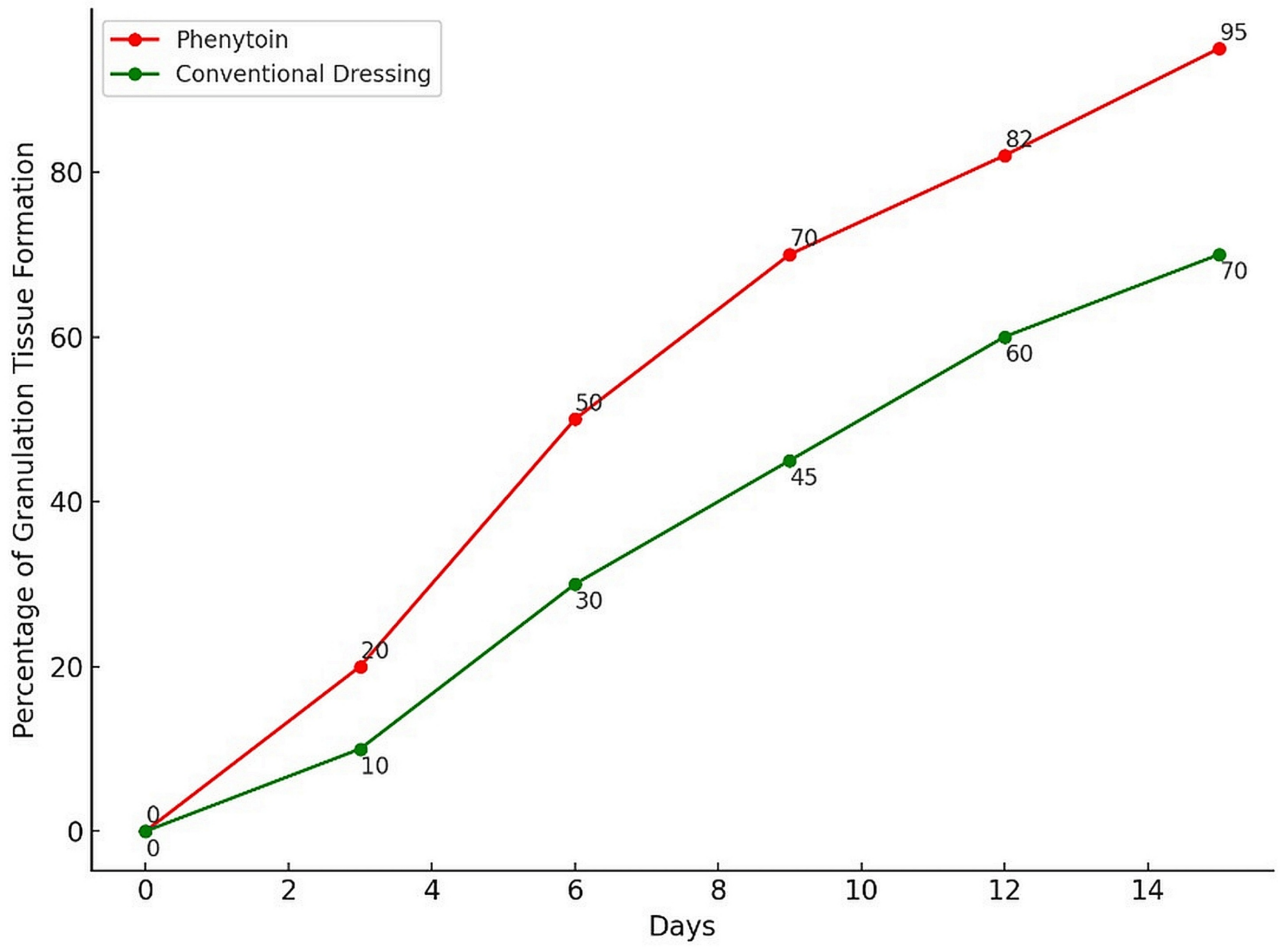
Progression of granulation tissue formation over time Granulation tissue formation is represented as a percentage

The reduction in the percentage of positive cultures to 14 (23.33%) in the phenytoin group and 10 (16.66%) in the conventional group by day 15 indicates that microbial control has been enhanced over time with both treatment modalities (Figures 1, 2).

The phenytoin group (P) shows earlier and faster formation of granulation tissue compared to the conventional dressing group (C).

## Discussion

This observational study revealed significant findings regarding the efficacy of topical phenytoin in the treatment of neuropathic DFUs with mild infections compared to conventional dressings. Our results demonstrated that the phenytoin group experienced a markedly higher rate of ulcer size reduction (>75%) and an earlier onset of granulation tissue compared to the conventional dressing group.

The superior performance of phenytoin in enhancing wound healing corroborates previous studies that highlighted its beneficial effects on wound management. Research has shown that phenytoin stimulates fibroblast proliferation and increases collagen deposition, which is crucial for the wound healing process [3,6]. For instance, a study by Prasad et al. indicated that topical phenytoin significantly improved wound healing metrics in DFUs, aligning with our findings of enhanced granulation and quicker ulcer size reduction [8]. Pendsey found that phenytoin reduced wound area more than controls; half of the phenytoin-treated cultures were negative by the seventh day, compared to 17% of the control group. A total of 72.5% of the phenytoin-treated ulcers healed fully, compared to 28.5% of controls [10]. Patil et al. found that phenytoin demonstrated efficacy as a topical agent, facilitating healing and effectively managing infections in DFUs [11]. A study conducted by Shaw et al. found no differences in DFU closure rates or DFU areas over time [12].

In contrast, the conventional treatment group in our study showed slower progress in wound healing, which is consistent with the general challenges associated with treating DFUs, such as prolonged inflammation and reduced neovascularization [2]. The delayed wound healing in conventional treatments can lead to higher risks of complications, as diabetic ulcers are prone to infections due to impaired immune responses. In a study conducted by Pai et al., the mean percentage reduction of ulcer area was found to be significantly greater compared to the control group (p < 0.05) [13].

The mechanism by which phenytoin accelerates wound healing can be attributed to its anti-inflammatory properties and its ability to modulate the wound healing phases. Phenytoin has been reported to reduce inflammatory cytokine expression while promoting keratinocyte proliferation, which is essential for the re-epithelialization phase of healing [4,5]. Additionally, its impact on collagen synthesis not only strengthens the new tissue but also ensures the structural integrity of the healed area, reducing the recurrence of ulcers.

Phenytoin has proven to be a highly cost-effective wound dressing, costing as low as 1-2 Indian rupees per patient, compared to 40-80 rupees for OpSite dressings, according to a study in 1993. Its usage not only reduces direct costs but also accelerates healing, with an average healing time of 6.2 days compared to 8.5 with OpSite [14]. From a clinical perspective, the findings suggest that topical phenytoin could be considered a viable alternative to conventional dressings for patients with DFUs, particularly those who are not responding well to standard care. The ease of application and the minimal side effects, primarily mild itching reported in only a small fraction of the phenytoin group, support its practicality in a clinical setting.

The retrospective methodology and lack of randomized control may add selection biases and restrict the generalizability of this study's encouraging outcomes. Prospective, randomized controlled trials should confirm these findings and examine phenytoin's long-term effects and safety in varied patient populations.

## Conclusions

This observational study assessed the efficacy of topical phenytoin in enhancing wound healing in neuropathic DFUs with mild infections, finding it more effective than conventional dressings. The results indicated faster size reduction and earlier granulation tissue formation. The study focused on selected cases of mild infections to provide a clear understanding of phenytoin's effectiveness. To confirm these promising findings, further randomized controlled trials are necessary to evaluate the therapeutic efficacy and safety of phenytoin across a broader patient population.
